# Experimental animal models for COPD: a methodological review

**DOI:** 10.1186/s12971-017-0130-2

**Published:** 2017-05-02

**Authors:** Vahideh Ghorani, Mohammad Hossein Boskabady, Mohammad Reza Khazdair, Majid Kianmeher

**Affiliations:** 1 Pharmaceutical Research Centre and Department of Physiology, School of Medicine, Mashhad University of Medical Sciences, Mashhad, Iran; 2 Neurogenic Inflammation Research Centre and Department of Physiology, School of Medicine, Mashhad University of Medical Sciences, Mashhad, 9177948564 Iran

**Keywords:** Chronic obstructive pulmonary disease, Emphysema, Animal models, Methods, Inflammation, Lung pathology, Airway responsiveness, Cigarette smoke

## Abstract

**Introduction:**

Chronic obstructive pulmonary disease (COPD) is a progressive disorder that makes the breathing difficult and is characterized by pathological conditions ranging from chronic inflammation to tissue proteolysis. With regard to ethical issues related to the studies on patients with COPD, the use of animal models of COPD is inevitable. Animal models improve our knowledge about the basic mechanisms underlying COPD physiology, pathophysiology and treatment. Although these models are only able to mimic some of the features of the disease, they are valuable for further investigation of mechanisms involved in human COPD.

**Methods:**

We searched the literature available in Google Scholar, PubMed and ScienceDirect databases for English articles published until November 2015. For this purpose, we used 5 keywords for COPD, 3 for animal models, 4 for exposure methods, 3 for pathophysiological changes and 3 for biomarkers. One hundred and fifty-one studies were considered eligible for inclusion in this review.

**Results:**

According to the reviewed articles, animal models of COPD are mainly induced in mice, guinea pigs and rats. In most of the studies, this model was induced by exposure to cigarette smoke (CS), intra-tracheal lipopolysaccharide (LPS) and intranasal elastase. There were variations in time course and dose of inducers used in different studies. The main measured parameters were lung pathological data and lung inflammation (both inflammatory cells and inflammatory mediators) in most of the studies and tracheal responsiveness (TR) in only few studies.

**Conclusion:**

The present review provides various methods used for induction of animal models of COPD, different animals used (mainly mice, guinea pigs and rats) and measured parameters. The information provided in this review is valuable for choosing appropriate animal, method of induction and selecting parameters to be measured in studies concerning COPD.

## Background

Chronic obstructive pulmonary disease (COPD) is a major cause of morbidity and mortality throughout the world and is characterized by chronic airway inflammation, mucus hypersecretion, airway remodeling, and emphysema, leading to reduced lung function and breathlessness [1–4]. Development of COPD is slow and progressive, with occasional exacerbations caused by inflammatory responses induced by triggering substances such as noxious gases, bacteria or viruses [2].

There are no effective treatments for COPD, because the mechanisms underlying COPD are poorly understood at the molecular level. The lack of a small-animal model that recapitulates the distinctive features of the disease in a certain time frame, is a major limiting factor in the study of COPD [1]. However, animal experimentation continues to provide approaches for treatment of all chronic diseases including those affecting the airways and lungs [5].

Animal models are used to study chronic obstructive pulmonary disease [6], investigate inflammatory processes [7] and determine the basic mechanisms of COPD [8]. Several animal species have been used as a model of COPD including rodents, dogs, guinea-pigs, monkeys and sheep [8] but an appropriate model highly depends on the aims of the study [9].

Different methods of induction of animal models of COPD, different animals used for this purpose and various measured parameters were comprehensively reviewed in the present article. Therefore, the present review will help investigators to choose an appropriate method for induction of an animal model of COPD and measure informative variables based on their study design.

## Methods

### Search strategy

A search for articles, published in English, from January 1967 to November 2015, was conducted using Google Scholar, PubMed and ScienceDirect databases. Overall, 221 relevant articles were identified from which, 70 articles excluded on the basis of the publication status, selected population and publication language. Therefore, 151 retrieved articles were eligible and were included in the review. The search terms included 5 keywords for COPD (chronic obstructive pulmonary disease, COPD, chronic bronchitis, emphysema, airway obstruction), 3 for animal model (animal model, animal experimentation, investigative techniques), 4 for exposure method (cigarette smoke (CS), lipopolysaccharide (LPS), elastase, combination inducers), 3 for pathophysiological changes (airway remodeling, airway inflammation, airway responsiveness) and 3 for biomarkers (biomarkers, cytokines, mediators).

### Inclusion and exclusion criteria

Articles were included if they: 1) provided different animal models of COPD; 2) provided sufficient and clear detailed method of animal exposure to inducers of COPD; and 3) evaluated parameters indicating the induction of an animal model of COPD. Abstracts or unpublished articles, human studies and non-English language articles were excluded.

### Management of search results

The search results were checked and included papers were reviewed by authors. We have presented information from each study and a qualitative conclusion was drawn.

## Inducers of COPD in various animal models

There are different approaches to imitate COPD in animal models [10]. These approaches include exposing laboratory animals to CS (the primary etiological factor for COPD), inflammatory stimuli (e.g., LPS), proteolytic enzymes (e.g., elastase), and genetic modification [7, 11]. In this section, different inducers of COPD that have been used in various animals are reviewed (Table 1).

**Table 1:** Different method used for induction of animal model of COPD and various measured parameters in each model

Induce.	Animal	Method	Measured parameters			Ref.
			TR	Path	Infl	
Cigarette	Mice	- 2 × 75 min/day, 5 day/week, for 1–12 weeks		✓		[1]
		- 2 × 30 min/day, 3 consecutive days, whole body exposure				[26]
		-12 Cig × 2 × 50 min/day, 5 day/week, for 8–24 weeks, whole body exposure		✓	C	[30]
		-150 ± 15 mg/m^3^ CS of TSP, 4 h/day, 5 day/week, whole body exposure				[27, 28]
		-150 mg/m^3^ CS of TPM, 3 h/day, 5 day/week, for 6 months				[136]
		- 5 Cig (12 mg of tar, 0.9 mg of nicotine, without filter) × 4/day, 30 min rest, 5 day/week, for 24 weeks		✓		[118]
		-250 and 500 mg/m^3^ CS of TPM, 2 × 50 min/day, 3 consecutive days, nose only and whole body exposure			M	[22]
		- 5/day, 5 day/week, for 6 months				[142]
		- 5 h/day, 5 days/week, for 6 months, whole body exposure		✓	C	[29]
		- 150 ± 15 mg/m^3^ CS of TSP, 4 h/day, 5 day/week, for 6 months				[143]
		- 12 Cig × 2 × 75 min/day, 5 day/week, for 1–12 weeks, nose only exposure		✓	C/M	[32]
		- 3 Cig (with filter), 5 day/week, for 6 months, nose only exposure		✓	C	[33]
		- 10 Cig (without filter), 512.6 mg/m^3^ CS of TPM, 2 × 35 ml puffs/min, 50 min/day, 5 day/week, for 22, 24, 45 days, nose only exposure			C	[34]
		- 3 Cig (12 mg of tar, 0.9 mg of nicotine)/day, 5 day/week, for 4 or 7 months	In vivo			[120]
		-103.36 ± 1.09 mg/m^3^ CS of TPM, 2 × 70- cm^3^ puffs/min, 6 h/day, 7 day/week, whole body exposure				[31]
	Rat	-8 Cig (14 mg of tar, 1.2 mg of nicotine, 15 mg of CO) × 2 × 30 min/day, 3–4 h interval between them, for first 2 weeks and 15 Cig (14 mg of tar, 1.2 mg of nicotine, 15 mg of CO) × 3 × 30 min/day, 3–4 h interval between them, from the third to the twelfth week		✓	M	[2]
		-12 Cig (10 mg of tar, 0.8 mg of nicotine, 10 mg of CO, with filter) × 3		✓		[37]
		-20 Cig × 2/day, 4–5 h interval between them, for 4 months		✓	C	[38]
		- 80–90 mg/m^3^ CS of TSP, 1 × 35 ml puffs (2-s duration)/min, 6 h/day, 3 day/week, for 3 days or 4 weeks or 12 weeks, whole body exposure		✓		[39]
		- 10 min/day, for 7 weeks				[40]
		-1 × 35 ml puffs (2-s duration)/min, 6 h/day, 3 day, whole body exposure			C	[41]
		-Cig (25 mg tar, 1.4 mg nicotine) × 2 × 1 h/day, 7 day/week, for 2, 4, 6, 8, 10, 12, 24, 36 weeks		✓	C	[42]
	Guinea pig	-1–5 Cig (5 mg nicotine, 6 mg tar, with filter), 2 × 20 ml puffs/Cig/min, 8–9 min/Cig/day, 10 min interval between cigarettes, 5 or 6 day/week, for 3 consecutive months, nose only exposure	In vitro	✓	C/M	[50–53]
		-1–5 Cig (5 mg nicotine, 6 mg tar, without filter), 2 × 20 ml puffs/Cig/min, 8–9 min/Cig/day, 10 min interval between cigarettes, 5 or 6 day/week, for 3 consecutive months, nose only exposure	In vitro	✓		[54–58]
		-4 Cig (5.3 ± 0.1 mg/l concentration of CS), 30 min/day, 7 day/week				[144]
		-10 Cig, 2 × 20 ml puffs/min, 8–9 min/Cig/day,10 min rest, for 1–60 days				[13, 145]
		- 10 Cig (without filter)/day,5 day/week, for1–12 months				[44]
		- 7 Cig (without filter)/day, 5 day/week, for 3–6 months				[13, 146]
		-5 Cig/day, 5 day/week, for 6 months				[13, 147, 148]
		- 2 h/day, 5 day/week, for 24 weeks				[13, 149]
	Dog	-2–7 Cig (0.27 mg tar, 3.25 mg nicotine, without filter)/day, 2 × 35 ml puffs/Cig/20 s, 7 day/week, for 2–4 months				[62]
		-12 Cig/day, 7 day/week, for 5 months				[150]
		-10 Cig (20 mg tar, 1.2 mg nicotine, without filter)/day, 2.5 h/day, 5 day/week, for 6 or 10 months				[59, 151]
	Monkey	- 6 h/day, 5 day/week, for 7 months		✓	C	[64, 65]
LPS	Mice	-IN 0.3 mg/kg of LPS, animals were killed 24 h after the challenge to LPS				[26]
		- IN 7 μg of LPS, 1 day/week, for 4 consecutive weeks		✓	C	[77]
		- intrapulmonary instillation, 0.5 mg/kg; volume =100 μL of LPS			M	[79, 80]
	Guinea pig	-IN 200 mL of LPS (5 mg/mL in sterile saline), twice/week, for 12 consecutive weeks		✓		[69]
		-IN 100 μL of LPS (10 mg/ml in sterile saline), twice/week, for 12 consecutive weeks		✓	C	[77, 82]
	Rat	- aerosolized LPS, 30 min/day		✓	C/M	[81]
Elastase	Mice	- IN 1.2 U of PPE, 1 day/week, for 4 consecutive weeks		✓	C	[77]
		- IT 2 U of PPE/100 g body wt in 100 μl saline				[95]
	Rat	- IT 28 U of PPE/100 g body wt				[96]
		- IT 55 U of PPE/100 g body wt in 0.5 ml saline		✓		[97]
		- IT 0.55 U of PPE/100 g body wt in 0.7 ml or 0.3 ml of 0.15 M NaCl			M	[98]
	Hamster	- IT 55 U of PPE/100 g body wt in 0.3 ml saline				[97]
		- IT 0.55 U of PPE/100 g body wt in 0.7 ml or 0.3 ml of 0.15 M NaCl			M	[98]
Combination Inducers	Mice	- IN 1.2 U of PPE, on day 1 and IN 7 μg of LPS on day 4, for 4 consecutive weeks		✓	C	[77]
	Rat	-2 × 30 min/day exposure to CS, for 2 days and on 3 day exposure to aerosolized LPS for 30 min; 5 h after LPS exposure, exposure to CS for 30 min			C/M	[81]

*IT* intra tracheal, *IN* intranasal, *Ref.* references, *Cig* cigarette, *h* hours, *Sec* second, *CS* cigarette smoke, *CO* carbon monoxide, *wt* weight, *LPS* lipopolysaccharide, *PPE* porcine pancreatic elastase, *TSP* total suspended particles, *TPM* total particulate matter, *TR* tracheal responsiveness, *Path* lung pathology, *Infl* inflammation, *C* cell, *M* mediators

## Cigarette-smoke (CS)

Tobacco smoking is the most important risk factor for COPD [2, 4] and the most common COPD-inducer employed in in vivo studies [12]. In addition, to mainstream cigarette smoke, environmental cigarette smoke may also contribute to respiratory symptoms and COPD [4]. Usually, standardized research-grade cigarettes should be used to easily deliver a specified dose of total suspended particles (TSP) or total particulate matter (TPM), including nicotine and carbon monoxide. These cigarettes are most commonly used in the University of Kentucky [13]. However, currently, there is no standardized method or protocol for animal exposure and this is one of the limitations of the use of CS as an in vivo COPD-inducer. Therefore, the type of cigarettes used to generate smoke (commercial vs. research cigarettes, with or with-out a filter), the constituents of the CS used for exposures, delivery systems (whole body vs. nose-only), and most significantly, the dose of smoke delivered to the animals are important determinant factors [7, 14]. Despite these limitations, CS has been shown to induce many features of COPD in animals, including pulmonary infiltration of macrophages and neutrophils, airway fibrosis and emphysema [9, 15–21]. A variety of animal species exposed to tobacco smoke to mimic COPD is described in this section [10, 13].

### Mice

These have been the most commonly used species exposed to CS for induction of animal model of COPD [7, 22]. Concerning the immune mechanisms, mice are the best choice as animal models of COPD. Furthermore, the murine genome has been greatly sequenced, and has shown similarities to human genomes [23]; Also, the possibility to manipulate gene expression are suggested [10, 13, 24, 25]. However, several studies have shown that different strains of mice show various levels of sensitivity to CS challenge [7]. Different exposure protocols were used in several studies and mice were exposed to CS once or twice/day, several times/week for various days/weeks/months in a smoking apparatus as whole body exposure [26–31] or nose-only exposure [32–34], (Table 1).

### Rats

Rats are also used as animal models of COPD [10, 24], but they are known as a poor model [16, 35], because these animals seem to be relatively resistant to development of COPD [10, 16, 24]. However, several studies have used rats because measurable emphysematous changes can be distinguished only 2 months after CS-exposure [13, 36]. Several studies showed that emphysema development in mice significantly differ from that in rats [24]. Different methods of induction of COPD in rats, based on exposure method and duration, and cigarette type [2, 37–42] are shown in Table 1.

### Guinea pigs

Guinea pigs are suitable species that are commonly used in COPD studies [12, 43–46]. These animals have many advantages as there are similarities between the anatomy and physiology of their lungs with those of humans [46–49]. In addition, various similarities in physiological processes, especially airway autonomic control and response to allergen, have been shown between guinea pigs and humans [16, 44, 46]. However, there are disadvantages such as lack of molecular tools, the need to test numerous compounds for pharmacological studies, and the cost of purchasing and keeping the animals [10]. Since, the advantages outweigh the disadvantages, guinea pigs are being applied in studies related to asthma and COPD [13, 46]. These studies indicated that guinea pigs develop COPD and emphysema-like lesions after a few months of exposure to active tobacco smoke [44] and physiological changes that mimic COPD in humans were showed in smoke-exposed guinea pigs [16, 44]. For example, in order to induce COPD in these animals, guinea pigs were exposed for 8-9 min to 5 cigarettes/day, 5 or 6 days per week for 3 consecutive months, with [50–53] or without filter [54–58], (Table 1).

### Canine (Dog)

Dogs have been extensively used as a model of asthma and COPD [59], because the pathology and pathophysiology of chronic bronchitis and emphysema after exposure to CS in dogs are similar to humans [59–61]. The canine model, similar to other models of COPD, has been used to examine new treatments before testing them in humans [59]. In a study, pulmonary fibrosis and emphysema were produced in dogs after direct inhalation of CS in a short period of time [62], (Table 1).

### Monkey

Another appropriate animal model for investigation of mechanisms underlying allergic airways diseases and COPD, is non-human primates. It was shown that monkeys exposed to CS, exhibit chronic respiratory bronchiolitis and other airway alterations [63]. Exposure to CS for 6 h/day, 5 days/week with a total suspended particulate concentration of 1 mg/m^3^, can generate experimental COPD in monkeys [64, 65], (Table 1).

## Lipopolysaccharide (LPS)

LPS instillation was shown to be able to induce a short-term model of COPD with some human features of the disease [6, 66]. LPS (a major component of the outer cell wall of Gram-negative bacteria) is present as a contaminant in CS, air pollution and organic dusts [9, 67–69]. LPS induces acute COPD exacerbations, when given either alone or concomitant with CS [16]. In addition, LPS may be important in bacterial infection-induced exacerbations of COPD, which contribute to the development of the disease [69, 70]. Chorionic exposure of animals to LPS has been shown to induce pathological features of COPD, such as pulmonary inflammation and airway hyperresponsiveness (AHR) as well as structural changes in the lung [9, 69, 71–76]. Inflammatory responses are induced after 12 weeks of twice-weekly LPS exposure [9, 76]. Several studies that used LPS for induction of COPD in animals are listed in Table 1.

### Mice

Investigations indicated that exposure of mice to inhaled LPS leads to emphysema-like changes which persisted for up to 4 weeks [74, 77]. In addition, it was reported that LPS is able to induce pathological and physiological changes of COPD such as AHR and increased airway inflammation in mice [77, 78]. In these studies, LPS was administered through intrapulmonary instillation using a MicroSprayer aerosolizer [79, 80] or via intranasal route [26, 77] (Table 1).

### Rats

In rat models, LPS can be inhaled in the same manner as mice resulting in pathological features of COPD [81], (Table 1).

### Introduction

Studies have demonstrated that LPS can induce COPD pathological features, similar to those of COPD patients, in guinea pigs. In these studies, guinea pigs were given intranasal instillation of LPS twice weekly, for 12 consecutive weeks [69, 77, 82], (Table 1).

## Elastase

Elastase is a proteolytic enzyme, which is released by activated neutrophils in the lungs and leads to breakdown of alveolar tissue and emphysema [9, 83]. The elastase model consists of instillation of elastolytic enzymes (such as the porcine pancreatic elastase (PPE), human neutrophilic elastase and papain) in the lung resulting in tissue damage and development of emphysema [9, 10, 84–86]. This model is used to induce inflammatory responses to initiate and perpetuate the inflammatory response seen in COPD [12, 87, 88]. The major advantages of the elastase model are the technical ease of inducing the disease by a single instillation of the enzyme in the lung and the ability to control the disease severity by adjusting the amount of enzyme [9, 16, 89, 90]. However, the disadvantage of the elastase model is that the function of elastase in COPD emphysema depends on several pathophysiological mechanisms which again brings up the number of clinical events [12, 91, 92]. A wide variety of animals has been used in the elastase emphysema model [16, 83, 93] to reproduce some characteristics of human CS-induced disease, such as augmentation of airspaces, inflammatory cell influx into the lungs, and systemic inflammation [84]. In some studies, to reproduce human pulmonary emphysema, intratracheal instillation of elastase was used in mouse models for more than three decades so, they are well characterized [94, 95]. Other studies used rats and hamsters to reproduce elastase-induced emphysema [96–98].

### Mice

A mouse model of COPD using elastase instillation could be produced by intranasal exposure to 1.2 units (U) of porcine pancreatic elastase 1 day/week for 4 consecutive weeks [77]. In some studies, mice received 2 U/100 g/BW porcine pancreatic elastase dissolved in 100 μl phosphate-buffered saline solution, intra-tracheally [95], (Table 1).

### Rats

Rats emphysema model was developed by injection of a single dose of intra-tracheal elastase (28 U/100 g/BW) and studied 7, 15, 30 and 365 days after injection [96]. In other studies, rats received a single intra-tracheal dose of elastase (55 U/100 g/BW dissolved in 0.5 ml physiological saline or 0.55 U/100 g/BW diluted in 0.7 ml or 0.3 ml of 0.15 M NaCl), [97]. In a study, Borzone et al. showed that 4 months after intra-tracheal instillation of a similar single dose of elastase, severe pulmonary emphysema with profound alterations in respiratory mechanics, were observed in hamsters [97, 98], (Table 1).

## Combination inducers

Animal models that mimic different aspects of inflammatory responses in COPD, could be developed by concomitant use of different inducers such as CS, LPS and PPE. For example, mice could be intranasally challenged with PPE and LPS for 4 weeks to induce COPD**-**like lung inflammation [77]. In another study, increased inflammatory response was observed following rats exposure to a combination of LPS and CS. In this model, rats were exposed to CS for 30 min twice a day for 2 days. On day 3 animals were exposed to LPS for 30 min and 5 h later, they were exposed to CS [81], (Table 1).

### Other models

Other agents have also been used to induce airway inflammation injury. The apoptosis model focuses on the failure of COPD lung to repair itself post-injury focusing on dis-regulated normal lung tissue turnover. Apoptosis-induced COPD has been linked to inhibition of VEGF receptors [12, 16, 99–101]. This model induces enlarged airspaces in a short period of time but does not affect the airways [12, 16]. In addition, genetically-altered models that mimic COPD, have been developed in recent years [10, 102, 103]. These models could be used for recognition of both physiological functions of different genes as well as possible mechanisms of COPD [10]. For example, emphysema and airspace enlargement can occur following exposure to CS, in gene-targeted mice [104]. However, currently, this model has been mostly restricted to proteinase “knockouts” in gene-targeted mice. These studies have uncovered an important role for macrophage elastases, particularly matrix metalloproteinase-12 (MMP-12) [105, 106], and a crucial role for neutrophil elastase [106, 107]. In this method, overexpression of interleukin 13 (IL-13), [108] and interferon gamma (IFNγ) was demonstrated [109], both leading to inflammation and airspace enlargement. IFNγ is also a marked element of structural cell apoptosis [106].

## Measured parameters

### Pathological changes

A main characteristic of COPD is airflow obstruction which is mostly irreversible. The airway obstruction may be the result of a combination of small airways narrowing, airway wall inflammation [10, 110] and emphysema-related loss of lung elastic recoil [1, 10, 111, 112]. Structural changes of the lung such as emphysema and small airway remodeling, are the pathologic features of COPD [113]. Small airway remodeling in COPD occurs via sub-epithelial fibrosis, mucus cell hyperplasia and in some cases, increase airway smooth muscle (ASM) mass [9, 113–117]. In addition, persistent infiltration of inflammatory cells such as macrophages, neutrophils, T and B-lymphocytes in the airway wall are features of airway remodeling [115–117] which could be caused directly by CS and LPS exposure of structural cells of the airway wall, independent of inflammation [9].

#### Mice

Chronic inflammation, increased cellular infiltration in the lung parenchyma, increased numbers of mucus-secreting goblet cells, thickening of airway epithelium and alveolar enlargement as well as airway remodeling in mouse models of CS-induced COPD were observed [1]. Also, in a mouse model of PPE and LPS-induced COPD, airway remodeling, lung inflammation, goblet cell hyperplasia, and alveolar enlargement were observed [77]. Similarly, emphysematous destruction, parenchymal inflammation, mucus hyper secretion and airway remodeling in a mouse model of COPD, were reported after CS exposure [29, 30, 32, 64, 118, 119]. In addition, significant increases in airway wall thickness and airspace size were observed after smoke exposure in mice [33, 120].

#### Rats

Increased bronchiole and arteriole wall thickness, bronchiole stenosis, increased alveolar size were shown in a rat model of COPD [2]. Lung function parameters such as airway resistance, respiratory system resistance, tissue damping, tissue elastance and respiratory system compliance increased in CS-treated rats [37]. In addition, elastase-treated rats showed mild airspace enlargement, fragmentation of alveolar spaces and inflammation [97, 121]. Elevation of neutrophils counts, mucus secretion, edema and lung inflammation in the lung and/or bronchoalveolar lavage were also seen in rat models of COPD [81]. In addition, an increase in airway wall thickness and airway narrowing, peribronchiolar inflammation, infiltration of large amount of inflammatory cells around the airways, enlargement of alveolar airspaces, destruction of septal walls of alveoli and pulmonary bullae as a morphological change seen in emphysema, were reported in a rat model of CS-induced COPD [38]. Damage of bronchial airway epithelium, neutrophil infiltration in the bronchial wall and epithelium were observed in rats following exposure to smoke [39]. Similarly, hyperplasia of bronchial epithelial cells, hypersecretion of mucus and development of peribronchial fibrosis were also found in rat models of COPD [42].

#### Guinea pigs

Baarsma et al. showed pulmonary inflammation and tissue remodeling, inflammatory cell influx, and enhanced small airway collagen content in LPS-induced guinea pig models of COPD [82]. Increased interalveolar septum, presence of lymphatic tissue in the lung parenchyma, destruction of alveolar wall, and existence of emphysema in the lung and intra-alveolar bleeding of lungs were also observed in guinea pig models of COPD following exposure to CS [50, 51, 54–57]. In another study, airway and parenchymal neutrophilia, increased goblet cell numbers, lung hydroxyproline content, airway wall collagen and airspace size, were reported [69]. Lung pathological changes in different animal models of COPD were summarized in Table 2.

**Table 2:** Lung pathology and tracheal responsiveness evaluation in animal model of COPD

Parameters		Animals	Methods	Ref
Lung Pathology		Mice	-Chronic lung inflammation, infiltration of cells in the parenchyma, mucosal secretion, ticking of airway epithelium, alveolar enlargement, airway remodeling, goblet cell hyperplasia	[1, 77]
			- Emphysematous destruction, parenchymal inflammation, mucus hyper secretion, airway remodeling	[29, 30, 32, 64, 118, 119]
			- Increased wall thickness and airspace size	[33, 120]
		Rat	- Increasing of bronchiole and arteriole wall thickness, bronchiole stenosis, increased alveolar size	[2]
			- Airway resistance, respiratory system resistance, tissue damping, tissue elastance, increased respiratory system compliance	[37]
			- Airspace enlargement, fragmentation of alveolar spaces, inflammation	[97, 121]
			- Elevation of neutrophils, mucus, oedema, lung inflammation in lung and/or bronchoalveolar lavage	[81]
			- Increased wall thickness, airway narrowing, peribronchiolar inflammation, inflammatory cell infiltration, enlargement of alveolar airspaces, destruction of septal walls of alveoli and pulmonary bullae	[38]
			- Bronchial airway epithelium injury, neutrophil infiltration	[39]
			- Hyperplasia of bronchial epithelial cells, hypersecretion of mucus, peribronchial fibrosis	[42]
		Guinea pig	- Pulmonary inflammation and tissue remodeling	[82]
			- Increased interalveolar septum, increased lymphatic tissue in lung parenchyma, destruction of alveolar wall, emphysema in the lung, intra-alveolar bleeding	[50, 51, 54–57]
			- Airway and parenchymal neutrophilia, increased goblet cell numbers, elevation of lung hydroxyproline content, increasing of airway wall collagen and airspace size	[69]
TR	In vivo	Mice	- Inhaled Mch: (6, 12, 25, and 50 mg/ml, 1 min, measuring Penh by WBPle after 10 min	[31]
	In vitro	Guinea pig	- TC preparation; Mch (10 ^-7^ to 10 ^-5^ mM) every 2 min, measuring EC_50_ using CRC	[52]
			- TC preparation; Mch (10 nM to 5 mM) every 2 min, measuring EC_50_ using CRC	[56]
			- TC preparation; Mch (10 ^-7^ to 10 ^-2^ mM) every 3 min, measuring EC_50_ using CRC	[58]
			- TC preparation; histamine (0.1 μM –10 mM) every 2 min, measuring EC_50_ using CRC	[54, 57]
			- TC preparation; isoprenaline (10 nmol/L to 100 μmol/L) every 2 min, measuring EC_50_ using CRC	[55]

*WBPle* whole-body plethysmograph, *Mch* methacholine, *TC* tracheal chain, *EC50* effective concentration causing 50% of maximum response (MR), *CRC* concentration response curve, *TR* tracheal responsiveness

## Tracheal responsiveness (TR)

Airway hyperresponsivness (AHR) is the main characteristic of asthma which also exists in COPD [54]. Moreover, tracheal responsiveness (TR) to different stimuli is observed not only in asthmatic animals but also in animals exposed to CS [122–126]. This parameter was assessed in some of COPD animal models, in vivo or in vitro.

### In vivo measurement of TR

In vivo evaluation of TR has been usually examined by measurement of enhanced pause (Penh) using whole-body plethysmograph after inhalation of increasing doses of methacholine (Mch) aerosol [31].

#### Mice

AHR was assessed by methacholine challenge and measurement of Penh using whole body plethysmography in CS-exposed mice. The main indicator of airway obstruction, measured as Penh, shows a strong correlation with airway resistance measured using standard procedures [31, 127] and was calculated from the chamber–pressure–time curve.

### In vitro measurement of TR

In several in vitro studies, tracheal responsiveness to Mch, histamine and isoprenaline was examined using cumulative concentrations-response curve of the corresponding agent and determination of EC_50_ [52, 54, 55, 57, 128, 129].

#### Guinea pigs

In a study, the tracheal muscle responses of a guinea pig model of COPD (induced by CS) to cumulative concentrations of histamine (0.1 μM to 10 mM) were measured. Then, cumulative concentrations- response curve was plotted and the effective concentrations of histamine causing 50% of maximum response (EC_50_ H) were calculated [54, 57]. In addition, concentration-response curves for isoprenaline in guinea pigs exposed to CS were also constructed by repeated administration of isoprenaline and EC_50_ was determined [55]. Similarly, tracheal responsiveness to Mch was also measured in tracheal smooth muscle by assessing the contraction induced by each concentration of Mch in proportion to the maximum contraction obtained by the final concentration of Mch in an animal model of CS-induced COPD [52, 56, 58]. Different methods of TR measurements in various animal models of COPD were summarized in Table 2.

## Inflammatory cells and mediators

### Total and differential white blood cell (WBC) counts

A variety of cell types is involved in the pathophysiology of COPD including neutrophils, macrophages, CD8-T-lymphocytes and eosinophils (which may play a major role in acute exacerbations of COPD). They release several inflammatory mediators and tissue-degrading enzymes which can orchestrate tissue destruction and chronic inflammation [10, 104, 130–135].

#### Mice

An increase in total cell number, mononuclear cells such as macrophages and lymphocytes (particularly CD8+ T cells) as well as neutrophils was shown in bronchoalveolar lavage (BAL) samples of mouse models of COPD [29, 30, 32, 34, 77, 136, 137]. Increased total inflammatory cell counts were reported in BAL of animal models of COPD which were mostly due to an increase in macrophages and neutrophils counts [33, 64].

#### Rats

Total inflammatory cells and neutrophils were increased in BAL of rat models of COPD [81]. Increased total leukocytes, macrophages, neutrophils and lymphocytes in BAL of rats after exposure to tobacco smoke were also reported [39, 41, 42].

#### Guinea pigs

Total WBC [51] and eosinophil counts in blood were increased in guinea pig models of CS-induced COPD [50, 53]. Total WBC, eosinophils and neutrophils in lung lavage of COPD guinea pigs were also increased [52]. In Table 3, a summary of total and differential WBC in the blood and lung lavage in different animal models of COPD was presented.

**Table 3:** Inflammatory cells and mediators in the blood and lung lavage of different animal models of COPD

Sample	Parameters	Animals	Methods	References
Blood	WBC	Guinea pig	- Total WBC	[51]
			- Total WBC and Eosinophils in blood	[50, 53]
	Mediators & Cytokines	Rat	- TNF-α, IL-8, IL-10	[2]
		Guinea pig	- IL-8	[50, 52, 53]
			- MDA	[50, 51, 53]
Lavage	WBC	Mice	- - Total cell and Macrophages, lymphocytes, neutrophils	[29, 30, 32, 34, 77, 136, 137]
			- Macrophages and neutrophils (Cells)	[33, 64]
		Rat	- Total leukocytes, macrophage, neutrophils and lymphocyte	[39, 41, 42]
			-dfd - Total cell and neutrophils in lung lavage	[81]
		Guinea pig	- Total WBC, eosinophils and neutrophils in lung lavage	[52]
	Mediators & Cytokines	Mice	- IL-8, TNF- α, IFN- γ	[118]
			- KC, TNF- α, MIP-2, MIP-1α, MCP-1 - TNF- α - IL-12, IL-4, CXCL-10, CCL-22	[22] [32] [79, 80]
		Rat	- TNF-α, IL-8, IL-10	[2]
			- Total protein	[81]
			- Total protein, TNF-α	[121]
			- Total protein, IL-6, IL-1β, TNF- α	[98]
		Guinea pig	- IL-8	[52]

*WBC* white blood cell, *TNF-α* tumor necrosis factor alpha, *ILs (IL-1β, IL-4, IL- 6, IL-8, IL-10, IL-12)* interleukin-1β, 4,6,8,10,12, *MDA* malondialdehyde, *IFN-γ* interferon gamma, *KC* keratinocyte chemoattractant, *MIPs (MIP-2 and MIP-1α)* macrophage inflammatory proteins, *MCP-1* monocyte chemoattractant protein-1

### Inflammatory mediators and cytokines

Several inflammatory mediators are involved in COPD pathogenesis [10, 138]. For example, macrophages secrete inflammatory mediators such as interleukin 8 (IL-8), tumor necrosis factor alpha (TNF- α), leukotriene B4 (LTB4) [135, 139], reactive oxygen species (ROS), monocyte chemotactic protein 1 (MCP-1) and elastolytic enzymes such as matrix metalloproteinase (MMP-2, MMP-9, MMP-12), and cathepsins K, L, and S in response to CS and other stimuli [140]. Also, neutrophils evidently contribute to COPD pathogenesis by secretion of serine proteases (neutrophil elastase, cathepsin G, proteinase) and metalloelastases MMP-8 and MMP-9 [135, 140]. In addition, IL-13, a Th2 cytokine has been proposed to be implicated in the pathophysiology of COPD [10, 141].

#### Mice

Duan et al. reported that the levels of IL-8, TNF-$\alpha$, and IFN-$\gamma$ in BAL of smoke-exposed mice were significantly increased [118]. Increased BAL inflammatory cytokine secretion such as keratinocyte chemoattractant (KC), TNF-α [84], macrophage inflammatory proteins (MIP-2 and MIP-1 α) and MCP-1 were also observed in animal models of CS-induced COPD [22]. Increased levels of ILs (IL-12 and IL-4) and chemokines (CXCL-10 and CCL-22) in BAL of LPS-exposed mice were also reported [79, 80].

#### Rats

Increased levels of TNF-α, IL-8, and IL-10 were seen in both serum and BAL of CS-exposed rats [2]. The levels of total protein in the BAL fluid were also significantly enhanced in rat models of COPD induced by a combination of CS and LPS [81]. In another study, the levels of TNF-α and total protein in the BAL fluid were elevated in rats with CS-induced COPD [121]. Similarly, total protein content and also some proinflammatory cytokines such as IL-6, IL-1β, and TNF-α in BAL were increased in rats after elastase treatment [98].

#### Guinea pigs

Increased serum levels of IL-8 and malondialdehyde (MDA) in guinea pigs model of COPD induced by CS, were reported [50, 51, 53]. In addition, Feizpour et al. indicated that the level of IL-8 in serum and BAL of guinea pig models of CS-induced COPD, were increased [52]. Inflammatory mediators and cytokines changes in the blood and lung lavage in different animal models of COPD were presented in Table 3.

## Conclusion

There is an enormous diversity of methods by which a study on COPD in animals can be done. Thus, there is a need for a standard protocol, which defines parameters to be evaluated and procedures (e.g. exposure procedure) to be followed. For development of an animal model representative of COPD, methods for induction of COPD, parameters for assessment and characteristics of human COPD should be assessed. In the present review, information regarding induction of experimental models of COPD in different animals, various methods used for this purpose, and different parameters that should be measured, was provided. This essential information is valuable for designing appropriate studies in future investigations on COPD.

## Abbreviations

AHR: Airway hyperresponsiveness
BAL: Bronchoalveolar lavage
Cig: Cigarette
COPD: Chronic obstructive pulmonary
CRC: Concentration response curve
CS: Cigarette-smoke
EC50: Effective concentration causing 50% of maximum response
IFN-γ: Interferon gamma
ILs: Interleukins
IN: Intranasal
IT: Intra tracheal
KC: Keratinocyte chemoattractant
LPS: Lipopolysaccharide
LTB4: Leukotriene B4
Mch: Methacholine
MCP-1: Monocyte chemotactic protein 1
MDA: Malondialdehyde
MIPs: Macrophage inflammatory proteins
PPE: Porcine pancreatic elastase
ROS: Reactive oxygen species
TC: Tracheal chain
TNF-α: Tumor necrosis factor alpha
TPM: Total particulate matter
TR: Tracheal responsiveness
TSP: Total suspended particles
VEGF: Vascular endothelial growth factor
WBC: White blood cell
WBPle: Whole-body plethysmograph
